# Daily Consumption of a Fruit and Vegetable Smoothie Alters Facial Skin Color

**DOI:** 10.1371/journal.pone.0133445

**Published:** 2015-07-17

**Authors:** Kok Wei Tan, Brigitte A. Graf, Soma R. Mitra, Ian D. Stephen

**Affiliations:** 1 School of Psychology, Faculty of Science, University of Nottingham Malaysia Campus, Semenyih, Selangor, Malaysia; 2 School of Biosciences, Faculty of Science, University of Nottingham Malaysia Campus, Semenyih, Selangor, Malaysia; 3 Food, Nutrition and Hospitality Group, Department of Food and Tourism Management, Hollings Faculty, Manchester Metropolitan University, Manchester, United Kingdom; 4 Department of Psychology, Faculty of Human Science, Macquarie University, North Ryde, Australia; 5 ARC Centre of Excellence in Cognition and its Disorders, Macquarie University, North Ryde, Australia; College of Tropical Agriculture and Human Resources, University of Hawaii, UNITED STATES

## Abstract

Consumption of dietary carotenoids or carotenoid supplements can alter the color (yellowness) of human skin through increased carotenoid deposition in the skin. As fruit and vegetables are the main dietary sources of carotenoids, skin yellowness may be a function of regular fruit and vegetable consumption. However, most previous studies have used tablets or capsules to supplement carotenoid intake, and less is known of the impact of increased fruit and vegetable consumption on skin color. Here, we examined skin color changes in an Asian population (Malaysian Chinese ethnicity) over a six week dietary intervention with a carotenoid-rich fruit smoothie. Eighty one university students (34 males, 47 females; mean age 20.48) were assigned randomly to consuming either a fruit smoothie (intervention group) or mineral water (control group) daily for six weeks. Participants’ skin yellowness (CIELab b*), redness (a*) and luminance (L*) were measured at baseline, twice during the intervention period and at a two-week follow-up, using a handheld reflectance spectrophotometer. Results showed a large increment in skin yellowness (p<0.001) and slight increment in skin redness (p<0.001) after 4 weeks of intervention for participants in the intervention group. Skin yellowness and skin redness remained elevated at the two week follow up measurement. In conclusion, intervention with a carotenoid-rich fruit smoothie is associated with increased skin redness and yellowness in an Asian population. Changes in the reflectance spectrum of the skin suggest that this color change was caused by carotenoid deposition in the skin.

## Introduction

Carotenoids are a group of red, orange and yellow plant pigments which are thought to be beneficial for human health. In the skin, carotenoids can act as antioxidants, potentially benefitting the reproductive and immune systems [1], and contributing to protection against the damaging effects of UV light [2,3]. Carotenoids cannot be synthesized *de novo* in the human body, so must be obtained from dietary sources, primarily from fruit and vegetables [4].

Dietary carotenoids can accumulate in human skin and generate measurable changes in skin color–particularly yellowness (CIELab b*) [5–7]. A number of previous studies have examined the impact of carotenoid supplementation—in the form of capsules or tablets—on skin color. Stahl and colleagues [5] found an increment in carotenoid levels in human skin after participants consumed capsules containing 25 mg of beta-carotene per day for 12 weeks. A similar effect was obtained with a supplement containing 24 mg (total) of three common dietary carotenoids (beta-carotene, lutein and lycopene) daily for 12 weeks. An increment in skin yellowness (b*) was observed after 6 weeks [7].

Correlational studies have examined the relationship between regular dietary fruit and vegetable intake and skin color [8]. Individuals with a higher estimated regular dietary carotenoid intake (based on analysis of food frequency questionnaire data) had a yellower (higher b*) skin tone [8]. Naturally increased fruit and vegetable intake over a six-week period has also been associated with increased skin yellowness (b*) and redness (a*)[9]. However, previous studies have not included control groups, exposing them to the possibility that confounding variables (such as increased sun exposure) may be contributing to the effect. Similarly, the impact of dietary intervention with fruit and vegetables (as opposed to supplementation tablets) on skin color has not previously been examined.

Most previous studies have focused on Caucasian populations, though Coetzee and colleagues [10] report that intervention with 15 mg beta-carotene capsules for 8 weeks had no measurable color change effect on the darker, sun-exposed facial and outer-arm skin of black Africans, though color change effects were found in photoprotected skin areas. To our knowledge, the impact of carotenoid intervention has not previously been investigated in an Asian population.

In the current study, we examine the impact of dietary intervention with a carotenoid-rich fruit and vegetable smoothie on the skin color of an Asian population in a randomized controlled trial.

## Methods

This study was conducted according to the guidelines laid down in the Declaration of Helsinki and all work was approved by the Faculty of Science Ethics Committee at the University of Nottingham Malaysia Campus. All participants gave both verbal and written prior informed consent.

### Participants

Eighty-one healthy Malaysian Chinese participants (34 males, 47 females; mean age 20.47, SD = 1.22) were recruited from the University of Nottingham Malaysia Campus. All participants were ethnically Chinese Malaysians. Exclusion criteria were: a) consumption of more than 3 cigarettes per day [2], b) use of any skin color alteration products (facial cleansing products were accepted, including those with UV protection, but participants who used bleaching and/or tanning products were excluded), and c) any chronic or acute illness, including malabsorption diseases, liver diseases, diseases of lipid metabolism or photosensitivity disorder.

Interested individuals attended an initial screening and intake meeting, where it was determined whether the participant met the specified inclusion and exclusion criteria, whether the participant was present on campus during the intervention period (February–April 2013) and was able to pick up their intervention product every weekday for the duration of the study.

### Study design

**Fig 1:**
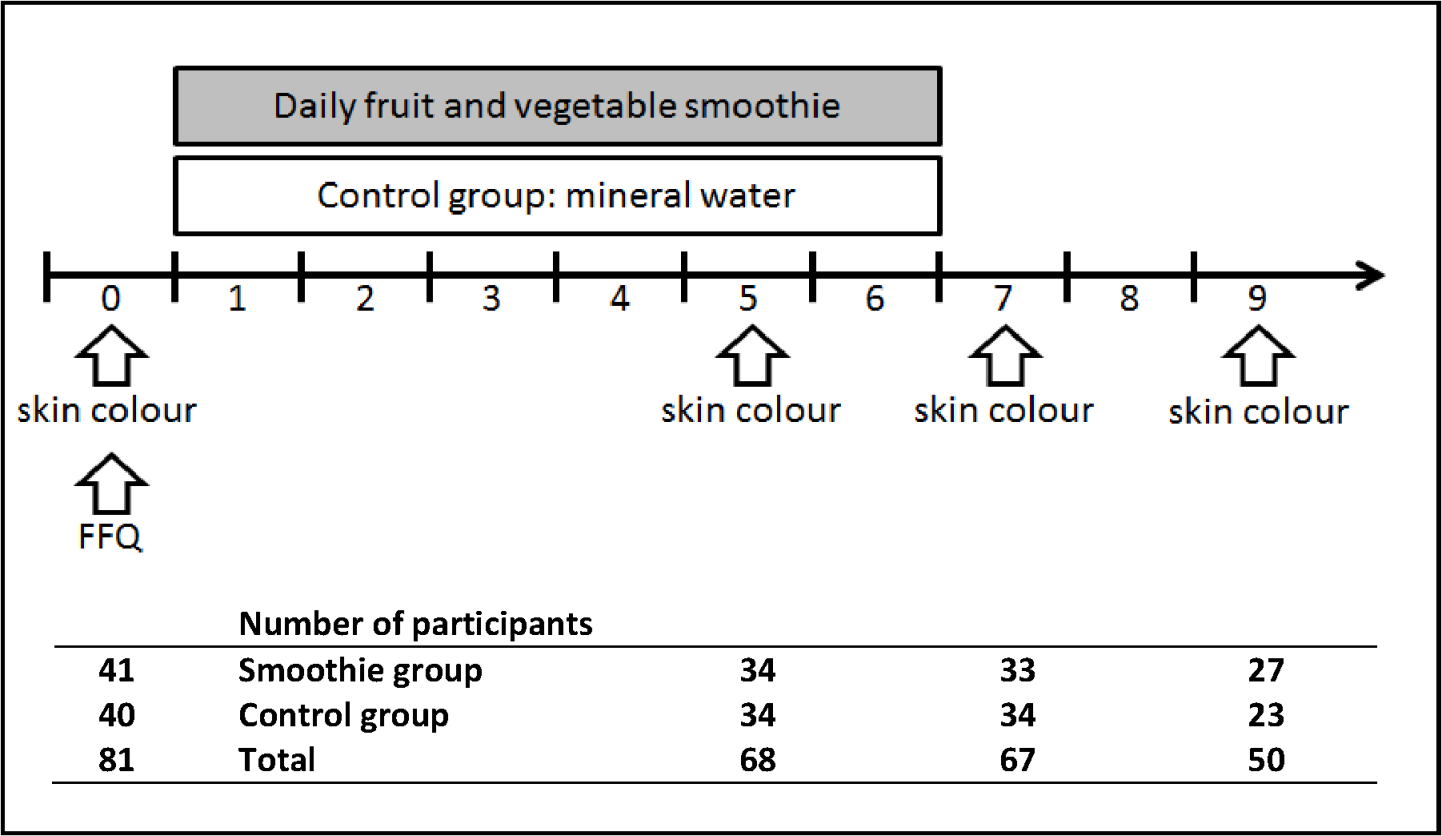
Study design and participant attrition. Considering total carotenoid and calorie intake in baseline diet, participants were assigned into intervention or control group by block randomization. Participants received either a smoothie or water every weekday for 6 weeks, and skin color was measured at baseline and in weeks 5, 7 and 9. 80.5% of the participants in the smoothie group and 85% of the participants in the control group completed the intervention.

This study follows a 2 (intervention vs. control group) x 3 (skin luminance, yellowness and redness) x 4 (sessions of measurement) mixed-model design. Participants were assigned to receive either the intervention product (a carotenoid-rich fresh fruit smoothie) or a placebo (bottled, filtered water described as “purified, detoxifying water”) every weekday for six weeks. Diet was measured at baseline, and body composition and skin color were measured four times during the study period (Fig 1).

### Dietary assessment and participant allocation

Participants completed an online food frequency questionnaire (FFQ) at baseline (within one week prior to beginning intervention). The FFQ was based on Loy et al. [11], and was modified to include a broader range of locally consumed fruits and vegetables: apricot, loquat, peach and persimmon (fruit); spinach, lettuce and water cress (green leafy vegetables); French beans and green peas (legumes); carrots and beetroot (root vegetables); peppers, broccoli and tomatoes (other vegetables) were added. For each item, participants indicated how frequently they consumed it (never, once a week, 2–4 times a week, 5–6 times a week, once per day, 2–3 times per day, 4–5 times per day), and the number of standard portions consumed per sitting. A photograph of a standard portion of the relevant food item was provided alongside each question in order to aid identification and to serve as a reference for standard portion size.

Total carotenoid and energy intake was estimated for each participant based on the “Nutrient Composition of Malaysian Foods” by Tee et al. [12]. Values for items not found in Tee et al. [12] were retrieved from the Canadian Nutrient File [13] (apricot, broad beans and loquat) or Fleshman et al. [14] (honey dew). Total daily carotenoid intake was calculated by multiplying the carotenoid content of one portion of each food item by the number of portions consumed per sitting, by the number of times the participant consumed that food item per week (where the response in the FFQ was a range of days, the mid-point was used), divided by 7 to give per day consumption, and finally summed across all food items to give an estimate of total daily carotenoid intake at baseline. A similar process was used to calculate daily energy intake.

Participants were pseudo-randomly assigned into the control or intervention group based on their baseline total carotenoid intake using the following procedure. Participants were ranked by carotenoid intake and randomly assigned in pairs, such that one of the two participants with the highest daily carotenoid intakes was randomly assigned to the intervention group and one to the control group, and so on, to ensure a balanced distribution of high and low carotenoid consumers in each group.

### Dietary intervention

Participants in the intervention group received a fresh fruit smoothie (500 mL), and control group participants received a bottle of filtered water (500 mL; described as “purified, detoxifying water”) every weekday for six weeks. Both groups were told that both the smoothie and the water have potentially beneficial effects on skin health. Six different smoothie recipes were created to deliver variations in flavor (Table 1). Each smoothie contained freshly prepared carrot juice (250 mL per smoothie), and locally grown tropical Malaysian fruit (kedongdong, chiku, pulasan, starfruit and red dragonfruit). Depending on availability of raw ingredients, recipes A–F were prepared on 9, 8, 2, 7, 7 and 4 of the days, respectively. Each smoothie contained 3% (w/w) lipids, in the form of milk, cream or oil to ensure optimal bioavailability of carotenoids from an aqueous food matrix [15], and lipids were provided in the form of milk, cream or oil. All fruit and vegetables were peeled and juiced with the exception of chiku, banana and red dragon fruit, where fruit pieces were blended with the mixed juices and the lipid source using a standard kitchen blender. After blending the mix thoroughly for 5 minutes the fresh smoothie was distributed to participants in reusable opaque plastic drinking bottles (to reduce possible photo-degradation of carotenoids; unpublished data suggests that carotenoid degradation in our smoothies in the bottles was negligible). Participants were asked to consume the smoothie within 3 hours of preparation. Participants from the control group collected five bottles of mineral water at the beginning of each week, and were asked to drink one bottle each weekday. Six different smoothie recipes (A–F) were created to deliver variations in taste. Energy content of the smoothie recipes was calculated using Tee et al. [12], the Canadian Nutrient File [13] and the manufacturer of Carotino [16].

**Table 1:** Composition of carotenoid rich smoothies. Participants in the intervention group consumed a carotenoid rich fresh fruit smoothie (500 mL) every weekday for 6 weeks. Fruit and vegetables were peeled and juiced with the exception of chiku, banana and red dragon fruit, where fruit pieces were blended with the mixed juices and the lipid source. Energy content (kcal) was calculated from values published in Tee et al [12], the Canadian Nutrient File [13] and Carotino’s manufacturer [16].

Ingredient	unit	smoothie A	smoothie B	smoothie C	smoothie D	smoothie E	smoothie F
Carrot juice	mL	250	250	250	250	250	250
Kedondong juice	mL	-	50	50	100	-	-
Lemon juice	tsp	-	-	15	15	-	-
Orange juice	mL	-	150	-	150	-	-
Pineapple juice	mL	-	50	-	-	-	-
Pulasan juice	mL	-	-	200	-	-	-
Starfruit juice	mL	100	-	-	-	-	50
Watermelon juice	mL	-	-	-	-	-	150
Banana	g	50	-	-	-	50	-
Chiku	g	-	-	-	-	50	-
Dragon Fruit (red)	g	50	-	-	-	-	-
Honey	tsp	-	-	-	15	-	-
Cream (30% fat)	mL	50	-	-	-	-	50
Milk (3.5% fat)	mL	-	-	-	-	140	-
Palm oil (Carotino)	mL	-	16.3	16.3	16.3	10	-
Energy	kcal	276	305	373	357	324	380

**Fig 2:**
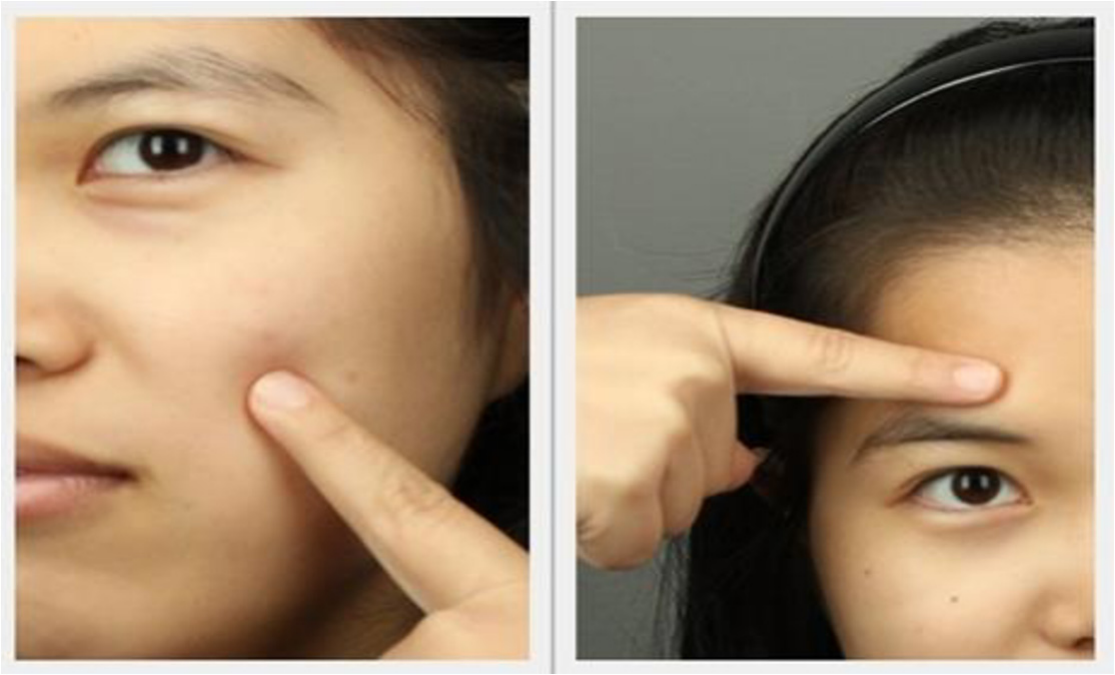
Skin color measurement of face regions. Locations of skin color measurements. Measurements were made from the left cheek, right cheek and forehead using a Konica Minolta CM2600d spectrophotometer. Measurements were made in the CIELab color space, in duplicate. Mean values across both measurements and all 3 locations were calculated for luminance (L*), redness (a*) and yellowness (b*) color axes. The individual in the image has given written informed consent (as outlined in the PLoS consent form) to publish her image.

Participants attended four measurement sessions during the study—baseline (week 0), mid-intervention (week 5), at the end of the intervention period (week 7) and two weeks after the intervention had ceased (week 9). A Konica Minolta CM2600D reflectance spectrophotometer (Tokyo, Japan) was used to measure skin color in CIELab color space–this has the advantage of taking accurate measurements in a perceptually uniform color space in which color axes are based on the opponent color properties of the human visual system, therefore representing color in a perceptually meaningful way. CIELab color space consists of luminance (L*), red-green (a*) and blue-yellow (b*) axes (see [17] for a detailed description). Color was measured at right cheek, left cheek and forehead (Fig 2). All measurements were conducted in duplicate, and the mean value of all measurements at the three face locations was taken as the measured value for analysis. Skin reflectance was measured at 10 nm intervals across from 360–740 nm (a range which encompasses the visual spectrum and extends slightly into the ultraviolet and infrared regions of the spectrum).

### HPLC-UV/VIS analysis of carotenoid content of smoothies

Representative smoothie samples were collected on all study days and stored at -20° C until analysis. Smoothie samples were freeze dried and carotenoids were extracted using ethanol/hexane (4:3, v/v) in 5 consecutive extraction cycles. Each smoothie sample was extracted and analyzed in triplicate. Extracts were kept at -20° C until HPLC-UV analysis. A Varian HPLC was used comprising of two solvent pumps (Varian 212-LC, CA, USA), a degasser (Degassit, MetaChem, Ludenscheid, Germany), an autosampler (Varian 410, CA, USA), and a UV-VIS detector (Varian 325, Agilent Technologies, Germany). Mobile phase A (MPA) consisted of methanol/water/ammonium acetate (1M) (97:3:0.2, v/v) and mobile phase B (MPB) consisted of MTBE. Carotenoids were separated on a Phenomenex Ultracarb ODS (30) HPLC column, with a length of 150 x 4.60 mm and 5 μm particle size (Phenomenex, CA, USA). Analysis was conducted at room temperature at a flow rate of 1 mL/min using a gradient from 0 to 10% MPB from 0 to 15 min, 10 to 50% MPB from 15 to 45 min, followed by a wash step consisting of 50 to 100% MPB from 45 to 50 min, 100 to 0% MPB from 50 to 51 min and an equilibration phase from 51 to 65 min at 0% MPB. Carotenoids were identified based on their co-elution with authentic standards. β-carotene was purchased from Nacalaitesque Inc. (Kyoto, Japan), while α-carotene and lycopene (and other carotenoids) were received from DSM (The Netherlands/Germany). Carotenoid quantification was based on absorption at 450 nm and calibration curves were prepared using standard solutions whose carotenoid content was precisely determined following quantitative spectrophotometry and the Beer Lambert law (as described in [18]). Varian Workstation version 6.9.3 was used to integrate carotenoid peaks from collected chromatograms.

### Body composition measurement

Participants’ height was recorded and body composition was measured using a Tanita SC-330 body composition analyzer (Tanita, Tokyo, Japan), which measures body weight and uses electrical impedance and entered height to estimate fat mass, fat-free mass, muscle mass, bone mass, total body water, body mass index, and basal metabolic rate.

### Data analysis

Of the eighty-one participants that were initially recruited (week 0), 71 attended the second measurement session (week 5). However, three participants were excluded from the analysis as their data were incomplete. Sixty-seven participants completed the intervention period and attended the third measurement (week 7). Of these, 50 participants came for the final follow-up measurement (week 9; Fig 1).

**Fig 3:**
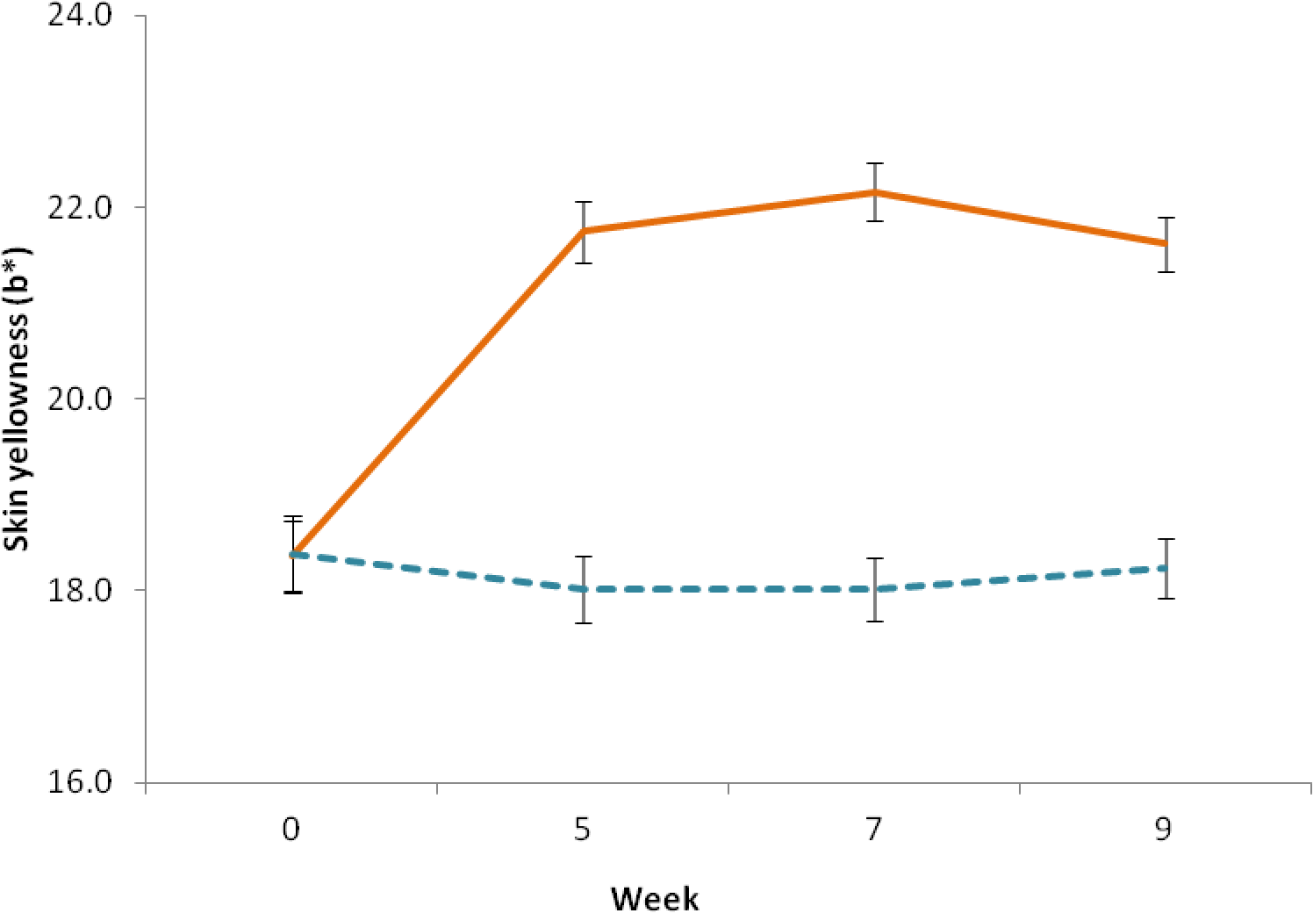
Change in skin yellowness. Skin yellowness was determined before (week 0), during (week 5), and at the end (week 7) of the intervention period, and at two week follow-up (week 9), using a handheld reflectance spectrophotometer. Solid line indicates the intervention group, dashed line indicates the control group, error bars represent the standard error of the mean.

To examine the changes in each color dimension over the course of the study, mixed ANOVAs were conducted (dependent variable: skin color (L*, a* or b*), within-subjects factor: measurement (baseline, second, third and fourth measurement), between-subjects factor: group (intervention vs. control group)). All pairwise comparisons were Bonferroni corrected. For the yellowness (b*) dimension, there was a significant main effect of group F(1, 48) = 44.127, p < .001, with participants in the intervention group exhibiting a yellower skin tone (EMM = 20.972, SE = .287) than those in the control group (EMM = 18.165, SE = .311). A significant difference in facial yellowness between measurements was found, F(3,121) = 43.916, p < .001 (Greenhouse-Geisser corrected), and an interaction between group and measurement was detected, F (3,121) = 62.38, p < .001. To investigate this result further, we conducted two separate repeated measures ANOVAs: one for the treatment group and one for the control group. A significant difference in skin yellowness between measurements was detected for the intervention group F(3, 78) = 106.773, p < .001, with increased skin yellowness at the second (mean difference = 3.382, SE = .239, p < .001), third (mean = 3.793, SE = .306, p < .001) and fourth measurement (mean = 3.257, SE = .239, p < .001) as compared to the baseline (mean = 18.36, SD = 1.68). No significant changes in skin yellowness were detected between the second, third and fourth measurements (p>.05). Also, no difference between measurements for the control group was detected F(3,66) = 1.103, p = .354 (Fig 3).

**Fig 4:**
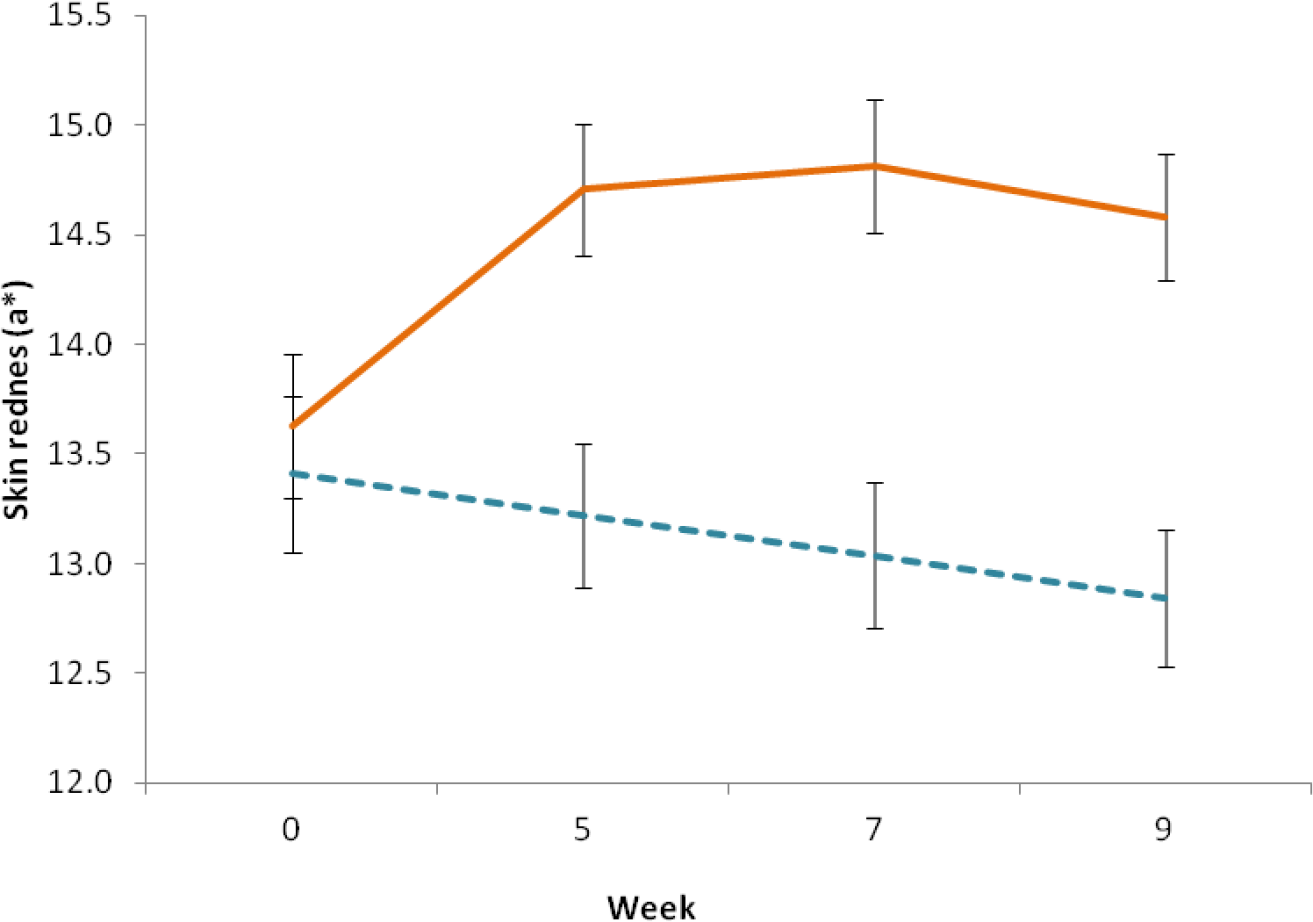
Change in skin redness. Skin redness was determined before (week 0), during (week 5), and at the end (week 7) of the intervention period, and at two week follow-up (week 9), using a handheld reflectance spectrophotometer. Solid line indicates the intervention group, dashed line indicates the control group, error bars represent the standard error of the mean.

A significant different in facial redness was found between the two groups F(1, 48) = 10.402, p = .002. Participants in the intervention group had overall redder skin tones (EMM = 14.431, SE = .274) as compared to those in the control group (EMM = 13.127, SE = .297). A significant difference in facial redness was found between measurements, F(2,112) = 3.115, p = .04, and an interaction between group and measurement was detected, F (2,112) = 9.937, p < .001. Separate repeated-measures ANOVAs showed a significant difference in skin redness between measurements for the intervention group F(3, 78) = 11.745, p < .001, skin redness increased in the second (mean difference = 1.077, SE = .239, p < .001), third (mean = 1.183, SE = .259, p < .001) and fourth measurements (mean = .953, SE = .234, p < .001) as compared to the baseline (EMM = 13.628, SE = .284). No significant difference was detected between the second, third and fourth measurements (p>.05). No significant difference in skin redness was found between measurements for the control group F(2, 40) = 2.006, p = .151 (Fig 4).

**Fig 5:**
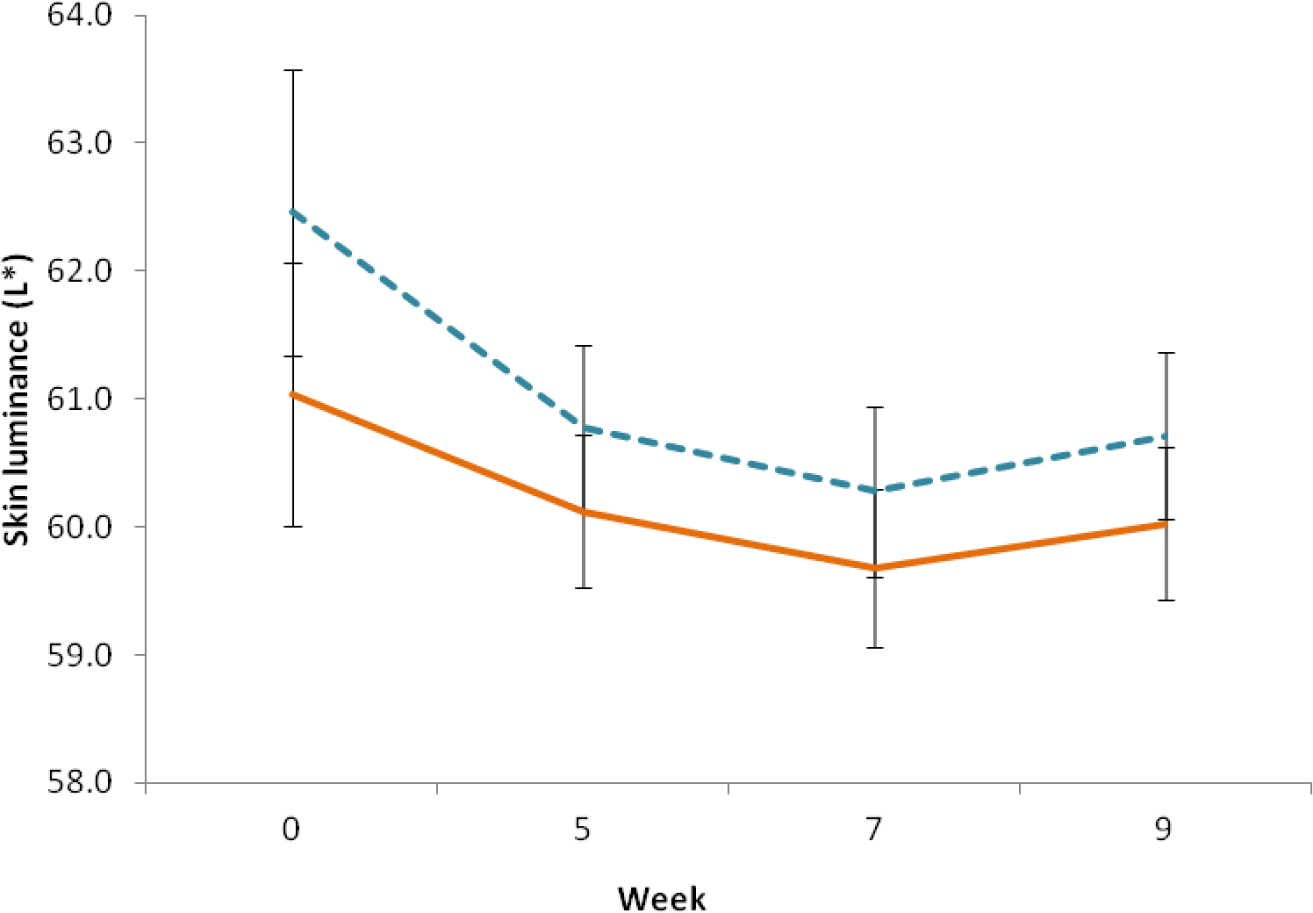
Change in skin luminance. Skin luminance was determined before (week 0), during (week 5), and at the end (week 7) of the intervention period, and at two week follow-up (week 9), using a handheld reflectance spectrophotometer. Solid line indicates the intervention group, dashed line indicates the control group, error bars represent the standard error of the mean.

No difference in skin luminance was detected between the smoothie and the control group, F (1, 48) = .862, p = .358. However, there was a significant change in participants’ skin lightness between measurements, F (2,79) = 5.031, p = .013. Participants’ skin was found to be darker at the third measurement (EMM = 59.98, SE = .455) as compared to the baseline measurement (EMM = 61.746, SE = .759; mean difference = -1.766, p = .037). No significant differences were found between other measurements (all p>.05). No interaction between group and measurement was found for skin lightness, F (2,79) = .328, p = .678 (Fig 5).

### Spectral analysis

**Fig 6:**
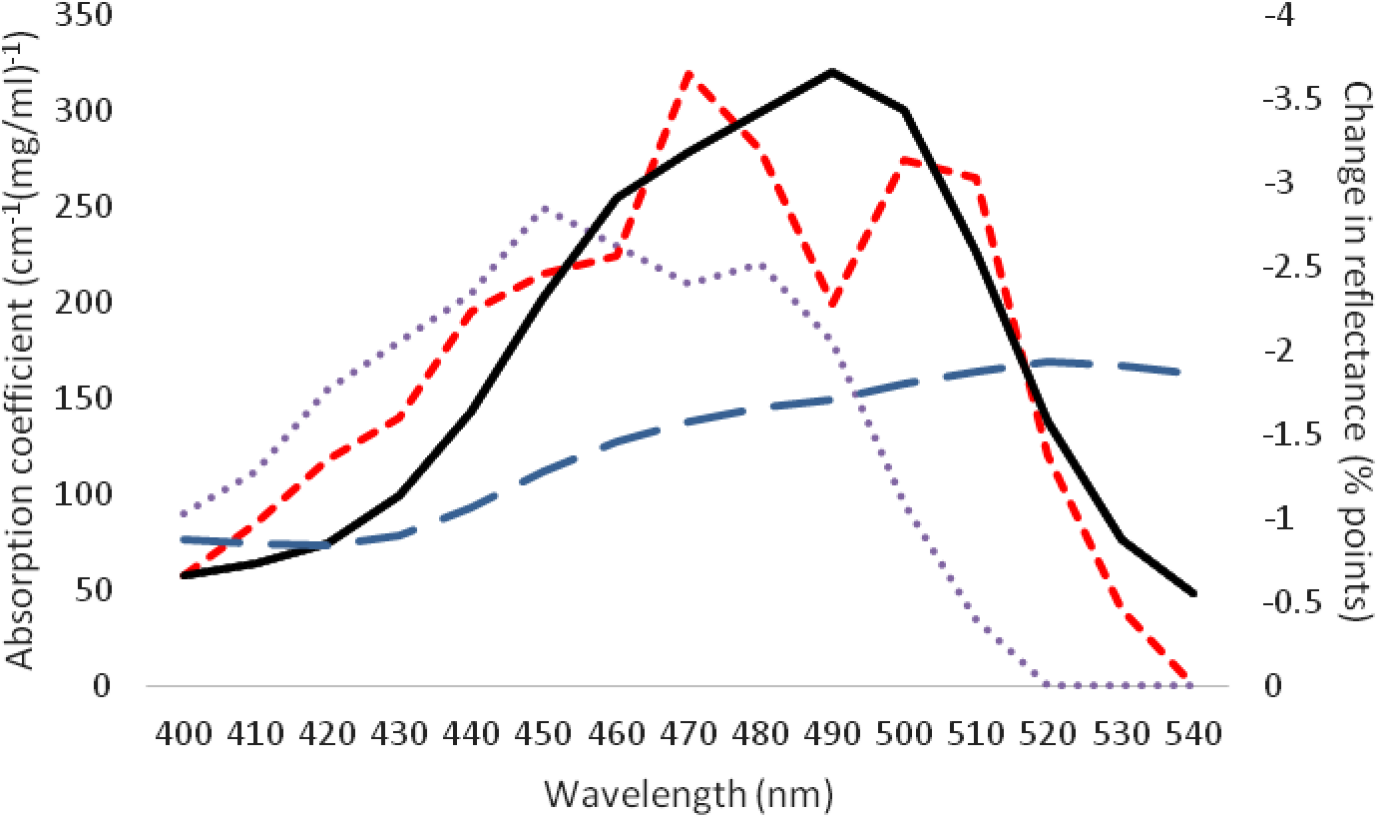
Changes in the reflectance spectrum of the skin. To confirm that color changes in the skin were likely to be correlated with carotenoid deposition in the skin, the change in the spectral reflectance of the skin between the baseline and week 5 measurements was calculated at 10 nm intervals from 400–540 nm. An increase of skin reflectance between 450 nm and 510 nm was seen in the intervention group (solid line), while no increased skin reflectance was measured in the control group (long dashed line). Absorption spectra for β-carotene (dotted line) and lycopene (short-dashed line) in 80% ethanol, 20% ether are included for comparison.

Spectral reflectance changes in the skin from baseline (week 0) to mid-intervention (week 5) were used to confirm that color changes in the skin were caused by carotenoid deposition [8,10]. β-carotene (a common yellow carotenoid) shows maximal absorbance in the 450–490nm range of the spectrum. Lycopene (a common red carotenoid) shows maximal absorbance in the 470–510nm range of the spectrum [19]. The intervention group exhibited reduced skin reflectance (equivalent to an increase in absorbance) in the 450–510nm range of the spectrum, suggesting deposition of both yellow and red carotenoids, rather than melanin (for which absorption peaks at ultraviolet wavelengths [20]) or hemoglobin (for which absorption peaks at shorter wavelengths than carotenoids [21]), in the skin. This pattern was not seen for the control group (Fig 6). This provides strong support for the idea that the color change observed in the supplementation group was due to the carotenoids in the smoothie, and not to other factors such as increased sun tanning or increased skin blood perfusion.

## Results

### Dietary and physiological characteristics of participants at baseline

The baseline diet of participants contained 2158 kcal per day (SD = 1309 kcal) and an estimated total carotenoid content of 6.7 mg (SD = 7.9 mg). Among the 151 food items listed in the Food Frequency Questionnaire, seven items contributed 76% of total carotenoids consumed: carrot juice (25.2%), spinach (21.9%), lettuce (9.1%), papaya (5.6%), kale (5.4%), green beans (4.7%) and carrot (4.1%). No difference in carotenoid (p = 0.844) or caloric (p = 0.264) intake was found between the intervention and control group. No significant difference in body composition or skin color was found at baseline between the intervention and control groups (Table 2).

**Table 2:** Physiological characteristics of participants at baseline. At baseline, participants’ height and weight were measured, and their body composition, total body water (TBW) and basal metabolic rate (BMR) estimated using a Tanita SC-330 body composition analyzer. Skin color was measured in the CIELab color space using a Konica Minolta CM2600d spectrophotometer.

	Intervention Group		Control Group		Differences	
Measurement	Mean	SD	Mean	SD	t	p
Height	167.27	8.75	164.18	8.80	1.59	.117
Weight	59.17	10.29	58.43	15.53	.23	.816
Fat Mass	11.52	4.40	14.31	8.95	1.66	.104
Fat Free Mass	47.65	9.77	44.11	10.44	1.45	.151
Muscle	45.06	9.36	41.67	9.93	1.46	.150
TBW	32.21	6.85	30.39	7.54	1.05	.297
Bone Mass	2.59	.42	2.44	.54	1.34	.184
BMR	5928.68	1057.48	5620.03	1225.36	1.12	.267
L	61.1	2.76	62.2	6.16	.94	.350
a*	13.6	1.43	13.64	1.77	.12	.906
b*	18.33	1.66	18.09	2.05	.54	.591

Independent samples t tests found no significant differences in body composition or skin color at baseline between the intervention and control group.

### Carotenoid content of smoothies

The daily amount of consumed smoothie (500 mL) contained on average 21.02 mg β-carotene (± 4.38 mg, ranging from 16.07 mg to 26.32 mg), 3.98 mg α-carotene (± 1.05 mg, ranging from 2.38 mg to 5.40 mg), and 1.24 mg lycopene (± 0.95 mg, ranging from 0 to 1.92 mg) (Table 3). On average a daily dose of 25.38 mg carotenoids was present in the freshly prepared smoothies, 80% was β-carotene, 15% was α-carotene and 5% was lycopene. Total carotenoid content (lycopene, α-carotene, and β-carotene) was highest in smoothies A and E, suggesting that ingredients other than carrot may have contributed to the total carotenoid content. The carotenoid contribution of red palm oil (Carotino), which was present in smoothie B, C, D and E was approximately 10% of the total in these recipes, and approximately 6% of the total across the study as a whole, despite the bright red color of red palm oil.

**Table 3:** HPLC analysis of carotenoid content of smoothies: Representative smoothie samples were collected every study day and stored at -20° C until analysis. Quantity of carotenoids was determined by HPLC-UV. Each smoothie sample was extracted and analyzed in triplicate. Six different smoothie recipes (A–F) were created to deliver variations in taste, and depending on availability of raw ingredients, smoothie A–F were prepared on 9, 8, 2, 7, 7 and 4 study days respectively. Values in the table are average carotenoid content in each of the 6 smoothie variations used in the study (± SD).

	smoothie A	smoothie B	smoothie C	smoothie D	smoothie E	smoothie F
β-carotene (mg)	26.32 ± 8.73	20.95 ± 4.03	16.07 ± 0.90	16.35 ± 3.70	25.65 ± 4.72	20.78 ± 2.49
α-carotene (mg)	5.40 ± 2.43	3.65 ± 1.12	2.38 ± 0.27	3.56 ± 0.65	4.74 ± 1.29	4.18 ± 1.33
Lycopene (mg)	-	-	0.57 ± 0.07	-	-	1.92 ± 0.37
total carotenoids (mg)	31.88 ± 10.98	24.60 ± 5.08	19.59 ± 0.94	19.76 ± 3.50	30.59 ± 5.56	26.68 ± 4.04

### Change in skin color over the course of the study

After four weeks of dietary intervention with a carotenoid rich fruit and vegetable smoothie, participants in the intervention group exhibited a significant increase in skin yellowness (Δb* = 3.257) (p<0.001) (Fig 3), but no change in skin yellowness was seen in the control group. Skin redness also increased significantly, though to a lesser degree (Δa* = 0.95) (p = 0.04) (Fig 4). This color change remained constant over the rest of the study period (measurement in week 7), and at the follow up measurement two weeks after the end of the intervention (measurement in week 9). The observed skin color modification coincided with a change in the reflectance spectrum of skin consistent with increased skin deposition of yellow and red carotenoid pigment (Fig 6), and not melanin or hemoglobin.

Skin luminance in both intervention and control groups decreased slightly (ΔL* = 1.73) over the course of the study period (p = 0.013) (Fig 5), i.e. no difference was seen between control and intervention group, when skin luminance was measured.

## Discussion

In line with previous findings that increasing carotenoid intake using dietary supplement capsules causes yellowing of the skin [5], we demonstrate that consumption of carotenoids in a fruit and vegetable based smoothie caused a change in skin color consistent with increased skin carotenoid content. This effect was seen only in the intervention, and not the control group. Further, the change in skin reflectance spectra is consistent with an increase in skin carotenoid content, rather than melanin or hemoglobin.

While previous studies have examined only the b* (yellowness) axis [2,5], we also examined the L* and a* axes. Participants in the intervention but not control group exhibited increased skin redness (a*). Carotenoids are a group of pigments that range in color from yellow to red [9], and we find that the change in skin reflectance is consistent with deposition of both yellow (β-carotene) and red (lycopene) carotenoids in the skin. This finding is consistent with previous evidence that consumption of fruit and vegetables is associated with increment in skin redness (a*) [9], and suggests that skin color changes are due to increased skin carotenoid deposition, and not sun tanning or skin blood perfusion. Further, our use of a control group that consumed filtered water instead of smoothie, and in whom the skin color changes were not observed, adds to our confidence that the skin color changes can be attributed to carotenoid consumption.

Deposition of carotenoids on the body surface has been widely reported for birds, where bright red and yellow carotenoid-based ornaments are displayed on the feathers (e.g. the red-winged blackbird, *Agelaius phoeniceus*, displays red epaulets based on red ketocarotenoids [22], or the yellow plumage of the greenfinch, *Carduelis chloris*, is based on yellow carotenoids [23]). The color of male birds’ plumage is thought to be related to the consumed component of carotenoids, their relative concentrations, and the total concentration of all carotenoids [24]. Since yellow carotenoids are known to be deposited in human skin, impacting skin color [2,5,8], it may be that the red carotenoid compounds also impact human skin color.

Other red-purple pigments may have been present in the fruits used to prepare the smoothie, for example betacyanin, a red-violet-colored pigment found in dragon fruit [25], though we are unaware of studies documenting the deposition of these pigments in human skin. Finally, it may be suggested that the increment of skin redness may also be due to increased blood perfusion in the intervention group, though we are unaware of any documented mechanism by which this may occur.

There was also a slight change in skin lightness between baseline measurement and the third measurement. However, this change also occurred in the control group, suggesting that it was not caused by the carotenoids contained in the fruit smoothie. Instead, we suggest that the slight decrement in skin luminance may have been due to an increase in skin melanin levels, which impacts skin reflectance across the visible and ultraviolet light spectrum [20], due to the increment of the exposure to sunlight during the duration of intervention. The third measurement was taken between the end of March and early April, at which time the temperature and amount of sunlight in Malaysia peaks [26].

While previous studies have found increases in skin yellowness (b*) associated with supplementation with carotenoid supplementation tablets [5,7,8], or with increased fruit and vegetable consumption in participants’ regular diets [8,9], the current study represents the first time that skin color changes have been associated with carotenoid supplementation using a fruit and vegetable smoothie as the vector of delivery. It is also the first study to our knowledge demonstrating an impact of carotenoid supplementation on skin color in an Asian sample. And this is the first study to our knowledge demonstrating an impact of carotenoid supplementation in a randomized controlled trial.

The findings of this study further strengthen the suggestion [2] that measuring skin color using a spectrophotometer can be a non-invasive technique to monitor levels of dietary carotenoid intake, and extends this possibility to an Asian population.

Insufficient intake of fruit and vegetables has been identified as among the world’s most significant causes of lost disability-adjusted life years [27], and is a significant problem in the developing as well as in the developed world [27]. Malaysians for example consume on average just 228 g of fruit and vegetables per day [28]–just 57% of the daily recommendation of 400 g. A desire to improve one’s appearance is an important motivator for healthy behaviors, especially in young people [29,30]. Facial skin carotenoid color has been shown to enhance the healthy (and therefore attractive [31]) appearance of human faces, and preliminary data suggests that, by showing participants how they could look if they increased their fruit and vegetable intake, individuals may be successfully motivated to increase their fruit and vegetable consumption [30]. Our results suggest that a carotenoid-rich smoothie may be an appropriate vector for the intervention of bioavailable carotenoids in a young Asian population.

Carotenoids’ antioxidant effect is thought to benefit the immune [32] and reproductive systems, [1] protect the skin from UV radiation [2,3] and to inhibit cell transformation and tumor growth in cancer, particularly when obtained from fruits and vegetables rather than supplements (see Schwartz [33] for a review). There is, however, some evidence that, under certain cellular conditions, high dose supplementation with carotenoid supplement tablets may also have a prooxidative effect, increasing cellular damage from ROS [34,35] and increasing the prevalence of cancer in smokers [36,37], though these adverse effects have not been reported from fruit and vegetable consumption. Further, there have been suggestions that high intake of fructose, such as that found in fruit juices, can cause weight gain and increases in LDL-cholesterol [38], though two recent meta-analyses suggest that these effects may not be attributable to fructose *per se*, but rather to increased caloric intake [39,40]. Nevertheless, since the smoothie recipes contained between 276 and 380 kcal, caution should be exercised to ensure that consumption of carotenoid-rich fruit smoothies takes place as part of a balanced diet.

### Conclusions

We have demonstrated for the first time that dietary supplementation with a carotenoid-rich fruit smoothie causes skin color changes in an Asian sample. In addition to increased yellowness, an increment in redness was also identified, suggesting that red carotenoid pigments (such as lycopene) may also be deposited in the skin. Analysis of the reflectance spectra of participants’ skin supported this hypothesis. Since appearance provides a powerful motivation for improved health behaviors, increased skin redness and yellowness have been shown to enhance healthy appearance in Caucasian samples, and a large number of people consume less than the recommended allowance of fruit and vegetables, intervention with a carotenoid-rich fruit smoothie may represent a viable mechanism for increasing fruit and vegetable intake, though this should take place as part of a healthy and balanced diet. Finally, this study suggests that skin color may serve as a non-invasive biomarker of fruit and vegetable intake.
